# Diatom diversity and distribution in Neotropical karst lakes under anthropogenic stress

**DOI:** 10.1371/journal.pone.0327201

**Published:** 2025-07-24

**Authors:** Margarita Caballero, Gabriela Vázquez, Ana Carolina Ruiz-Fernández, Javier Alcocer, Lucy Natividad Mora-Palomino

**Affiliations:** 1 Laboratorio de Paleolimnología, Instituto de Geofísica, Universidad Nacional Autónoma de México, Ciudad Universitaria, Ciudad de México, México; 2 Instituto de Ecología, A.C., Xalapa, Veracruz, México; 3 Instituto de Ciencias del Mar y Limnología, Universidad Nacional Autónoma de México, Unidad Mazatlán, Sonora, México; 4 Grupo de Investigación en Limnología Tropical, Facultad de Estudios Superiores Iztacala, Universidad Nacional Autónoma de México, Tlalnepantla, México; 5 Instituto de Geología, Universidad Nacional Autónoma de México, Ciudad Universitaria, Ciudad de México, México; Universitat de Barcelona, SPAIN

## Abstract

Lake degradation is an important environmental problem in the Neotropics, where rapid population growth is leading to increasing human impact. However, baseline studies in these lakes are still scarce. This study focussed on the hydrochemistry, trophic status and analysis of diatom diversity and ecological distribution in Neotropical karst lakes in southern Mexico, presenting a high-resolution paleolimnological reconstruction of changing hydrochemical and trophic characteristics since the late 1950s based on multiproxy analysis of a sequence dated by ^210^Pb and ^14^C. Four of the sixteen freshwater lakes had higher salinity (300−500 mg L^-1^), sulphates, turbidity and eutrophic conditions. These impacted, eutrophic to hypertrophic ecosystems receive soil-derived sediment, organic matter, urban and agricultural effluents through river inflow. Two diatom taxa were characteristic of these impacted lakes (*Aulacoseira granulata* var. *angustissima* and *Stephanocyclus meneghinianus*) while eight were preferentially present in the lower-salinity, oligo-mesotrophic lakes. Some of the lower-salinity taxa have restricted Neotropical karst distributions (*Cyclotella petenensis, Discostella* sp*., Mastogloia calcarea* and *Planothidium* sp.), and are in danger of local extirpation as hydrochemical changes and eutrophication increase. The beta diversity (βw) of the full data set was low (2.6), driven mostly by the hydrochemical and trophic status differences between the four most impacted lakes and the rest. Paleolimnological analysis allowed us to determine that the currently impacted lakes previously had lower salinity and trophic conditions, comparable with the currently non-impacted lakes. In Lake Peñasquito, increasing erosion during the 1970s was associated with the first appearance (1980s) and gradual increase of diatom taxa characteristic of lake degradation, and a critical transition point was observed in the diatom assemblages by ca. 2008.

## Introduction

Southern Mexico is a highly biodiverse region of priority for conservation as it holds some of the country’s last remnants of tropical rainforest, developing over fragile, karstic, thin-soils [1,2]. The karstic province in southern Mexico is part of the tropical karst belt [3] that includes the Caribbean region, inclusive of the Florida and Yucatán peninsulas. However, even though several national parks and protected areas have been established, human activities such as logging, agriculture, grazing, and wastewater discharges, have increasingly altered the landscape of the region, with important losses of forested areas during the last few decades [4,5]. Basin-wide degradation driven by human activities does not only affect the terrestrial ecosystems, but also the aquatic environments. Landscape degradation frequently leads to lake eutrophication, which is one of the most common degradation process in lakes worldwide [6]. Not surprisingly, there have been reports of increasing turbidity and water-colour changes in some of the lakes, for example in the touristically attractive Montebello Lakes region in Chiapas [7]. However, as is the case for many northern hemisphere Neotropical karst regions, degradation processes in the Montebello Lakes are difficult to evaluate because little is known about their limnology, biodiversity and possible response to environmental changes. In these lakes very few limnological studies were done before 2010 and therefore there is not a baseline or reference condition for these ecosystems.

Amongst the most useful tools to evaluate environmental and ecological change in lakes are modifications in their algal communities. Specifically, diatoms are used for environmental assessments because their siliceous valves can be preserved in the sediments and changes in their associations along stratigraphic sequences allow to assess the present condition of a lake and its recent history [8]. Nevertheless, this approach is limited by the deficient knowledge of diatom diversity and ecological preferences in northern hemisphere Neotropical karst lakes. To improve diatom-based paleolimnological assessments documenting recent ecological change processes in this kind of lakes it is necessary to study the diatom associations in surface sediments at sites with contrasting characteristics. In this study we explore diatom diversity and ecological distribution in 16 Neotropical karst lakes located in or near natural protected areas in southern Mexico: the “Naha-Metzabok” Flora and Fauna Protection Area, and the “Lagunas de Montebello” National Park. This study aims to contribute to the knowledge of diatom biodiversity in the northern hemisphere Neotropical karst regions and their distribution along environmental gradients. Specifically, we aim to identify the species with the highest abundance and frequency of occurrence (highest regional occupancy) and those that could be used as indicators of anthropogenic degradation processes. We also aim to use this information to investigate how anthropogenic stressors have affected these lakes in the last decades, by using titanium (Ti) and diatom-based paleolimnological analysis in one of the currently eutrophic and turbid lakes (Lake Peñasquito).

## Methods

### Site description

**Fig 1:**
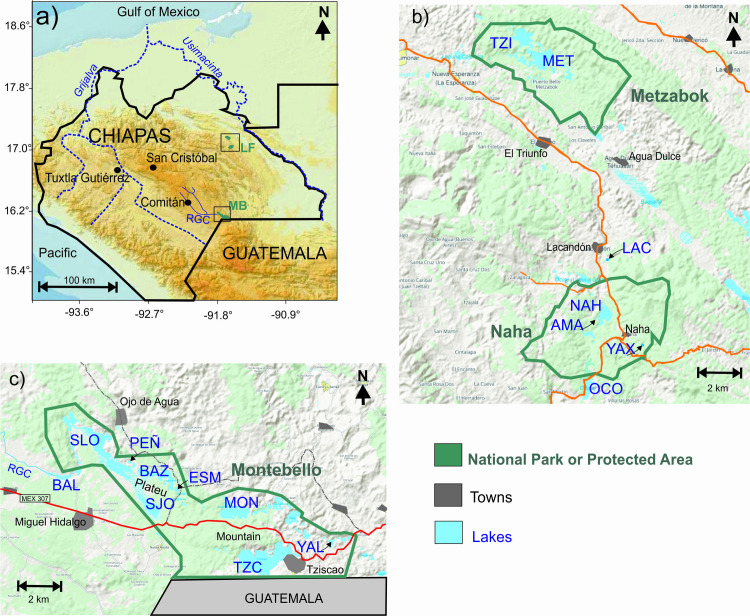
Location of the studied lakes in southern Mexico (Modified from public domain topographic maps from the Instituto Nacional de Geografia y Estadística (INEGI) at https://gaia.inegi.org.mx/mdm6/ and https://www.inegi.org.mx/temas/topografia/). 1a) Southern Mexico, with the location of the Naha-Metzabok protected areas in the Lacandona Forest region (LF) and the “Lagunas de Montebello” National Park (MB). 1b) Lacandona forest region with the location of the Naha-Metzabok protected areas (green polygons) and the studied lakes (TZI = Tzi-Bana, MET = Metzabok, LAC = Lacandon, NAH = Naha, YAX = Yaxha, OCO = Ocotalito). 1c) The Montebello Lakes region, with the location of the “Lagunas de Montebello” National Park (green polygon), the Río Grande de Comitán (RGC) and the studied lakes (BAL = Balamtetik, SLO = San Lorenzo, PEÑ = Peñasquito, BAZ = Bosque Azul, ESM = Esmeralda, SJO = San Jose, MON = Montebello, TZC = Tziscao and YAL = Yalalush).

This study included surface sediment samples from 16 lakes within the Grijalva-Usumacinta aquatic ecoregion in southern Mexico [9], which drains to the Gulf of México (Fig 1a). One of these lakes (Lake Peñasquito) was also selected for paleolimnological analysis. The lakes are located on folded Mesozoic to Cenozoic limestones of the Chiapas highlands, where fengcong-cockpit tropical karst [3] is dominant. They are located on mountainous terrain and range in altitude between 540–1500 m asl. They originated by dissolution and can be classified either as dolines (sinkholes), uvalas (coalescence of sinkholes) or poljes (elongated, flat-floored depressions). They have a large depth range (Z_max_), from 2.6 to 86 m, and vary largely in surface, from around 1–300 ha (Table 1). The smaller lakes are usually dolines (Peñasquito, Yalalush, Lacandon, Amarillo and Yaxha), while the larger are poljes (San Lorenzo and Tziscao).

**Table 1:** Main characteristics of the studied karstic lakes in southern México.

	Montebello Lakes: Plateau					Montebello Lkes: Mountain				Lacandón Forest: Metzabok			Lacandón Forest: Naha			
Lake	Balamtetik	San Lorenzo	Bosque Azul	Peñasquito	San Jose	Esmeralda	Yalalush	Tziscao	Montebello	Tzí’Bana	Metzabok	Lacandon	Amarillo	Naha	Ocotalito	Yaxha
**Code**	BAL	SLO	BAZ	PEÑ	SJO	ESM	YAL	TZC	MON	TZI	MET	LAC	AMA	NAH	OCO	YAX
**Lae Type**	U	P	U	D	U	D	D	U	P	U	U	D	D	U	P	D
**Latitude**	16°7’	16°9’	16°7’	16°7’	16°6’	16°6’	16°5’	16°5’	16°6’	17°7’	17°7’	17°0’	16°59’	16°59’	16°57’	16°58’
**Longitude**	91°47’	91°44’	91°46’	91°45’	91°44’	91°43’	91°38’	91°40’	91°42’	91°38’	91°37’	91°35’	91°35’	91°35’	91°36’	91°34’
**Altitude (m)**	1461	1462	1462	1426	1463	1463	1452	1488	1500	542	542	545	830	830	905	930
Area (ha)*	13.6	181.3	52.5	4	60.6	1.1	11.5	306.6	96.2	88	125	0.9	1.9	59	38	3.6
**Z**_**max**_ (m)*	3	67	58	46	30	7	23	86	45	51	20	2.6	9.5	23	21	34
**T**_**sup**_ **(°C)**	21.9	23.2	21.6	23.5	22.0	23.0	21.1	22.3	22.1	28.3	30.0	26.3	27.6	26.4	27.0	27.0
**T**_**bott**_ **(°C)**	21.4	20.8	17.6	18.2	21.9	22.3	20.7	18.4	18.8	21.0	26.0	26.0	21.4	21.2	20.0	21.4
**DO**_**bott**_ **(mg L**^**-1**^)	0.8	0.4	0.0	0.1	6.2	5.1	4.0	0.0	0.0	0.1	4.0	5.6	9.0	0.6	0.2	0.2
**pH**	7.5	8.3	8.3	7.6	8.6	7.4	7.9	9.2	8.8	7.6	7.8	7.6	8.34	7.7	7.7	7.8
**K**_**25**_ **(µS cm**^**-1**^)	587	382	362	364	293	358	234	225	155	296	260	198	201	322	215	185
**TDS (mg L**^**-1**^)	500	339	324	299	93	275	158	187	156	207	200	156	156	200	158	150
**SO**_**4**_^**—**^**(meq L**^**-1**^)	2.85	2.33	1.49	2.06	0.25	0.16	0.01	0.09	0.08	0.19	0.20	0.07	0.04	0.02	0.03	0.03
**%SO**_**4**_^**--**^	36.2	46.2	33.8	43.8	15.8	4.0	0.5	3.1	3.2	4.3	4.2	2.1	1.1	0.3	0.9	0.9
**Z**_**SD**_ **(m)**	0.3	0.5	1.1	2.5	1.8	5.0	3.4	8.1	6	2.1	3.6	0.9	0.9	2.9	2.1	7.0
**Chl *a* (mg m**^**-3**^)	63.3	31.7	31.3	17.3	4.8	5.5	4.0	0.4	0.5	2.9	0.2	30.7	32.1	7.0	8.3	6.2
**DIN (µM)**	76.6	13.5	3.4	11.6	16.1	6.6	32.9	2.0	1.6	8.0	6.3	12.5	7.8	9.0	15.0	5.7
**TP (µM)**	9.2	3.0	4.8	4.1	1.4	1.3	7.6	5.0	4.0	1.1	6.0	1.4	1.2	1.0	1.3	1.1
**SRP (µM)**	6.9	1.2	0.1	0.9	0.8	1.0	0.8	0.01	0.02	0.7	0.7	0.6	1.0	1.0	0.7	0.6
**SRSi (µM)**	293	198	68.3	89.2	11.7	55.3	23.3	11.5	2.6	42.4	42.4	28.1	11.7	39.0	48.6	6.7
**DIN/ TP**	8	5	1	3	11	5	4	0	0	7	1	9	6	9	12	5
**DIN/ SRP**	11	12	42	13	20	7	40	198	80	12	9	21	8	9	22	10
**SRSi/ DIN**	4	15	20	8	1	8	1	6	2	5	8	2	1	4	3	1
**SRSi/ SRP**	43	172	854	96	14	58	28	1150	130	65	61	46	12	41	71	12
**TSI**	74	69	64	59	54	50	53	37	39	52	42	65	64	53	55	47
^**0**^ ***D***	12	16	32	26	15	21	28	24	15	18	21	16	–	13	16	17
^**1**^ ***D***	7.6	5.5	15.4	14.2	5.5	11.3	6.8	11.9	6.8	5.7	11.3	8.2	–	3.7	5.2	3.9
^**2**^ ***D***	5.5	3.7	10.2	10.2	3.6	8.1	3.4	7.6	5.1	3.5	8.3	5.2	–	2.1	3.3	2.5

U = uvala, D = doline, P = polje, T_sup_. = surface temperature, T_bott_. = bottom temperature, DO_bott_ = bottom water dissolved oxygen concentration, K_25_ = electrical conductivity, TDS = total dissolved solids, Chla = chlorophyll *a*, DIN = dissolved inorganic nitrogen, TP = total phosphorus, SRP = soluble reactive phosphorus, SRSi = soluble reactive silica, TSI = Lamparelli’s trophic state index, ^0^*D* = Species richness, ^1^*D *= Shannon diversity, ^2^*D* = Simpson diversity. *Data for Balamtetik, San Lorenzo, San José, Bosque Azul, Esmeralda, Montebello, Tizsicao and Yalalush from Alcocer *et al.* 2016, for the rest of the lakes they correspond with field measurements and estimates in Google Maps.

The climate of the Chiapas highlands ranges from tropical-humid in the lower altitude areas to temperate-humid in the higher altitudes, with precipitation concentrated between June and October. Tropical-humid climates dominate in the lower altitude LF (~500–900 m asl), with mean annual temperature of ~22°C and precipitation of ~ 2000 mm yr^-1^, while temperate-subhumid to temperate-humid climates are present in the higher altitude MB region (~1,500 m asl), with mean annual temperature of ~18°C and precipitation that ranges from 900 to 2500 mm yr^-1^. Vegetation transitions according to altitude [10], from evergreen tropical rainforests (usually < 1,000 m asl) to cloud forests (~1,000–1,300 m asl) and mixed pine-oak forests (usually > 1,000 m asl). The vegetation is a mosaic of the three associations, however in the lower altitude LF evergreen tropical forests and cloud forests are dominant while in the higher MB region cloud forests and pine-oak forests are most abundant.

Seven lakes (Fig 1b) are in the Lacandona Forest (LF), a region with a lower population density and agricultural activity compared to the Montebello Lakes (MB) region, therefore these are mostly low-human impact lake systems. Nine lakes are in the MB region (Fig 1c) and they include six plateau lakes [11], located on the flatter NW section of the MB national park, where human impact is most intense. Previous work has identified that the interconnected plateau lakes are turbid and with a higher trophic status compared with the rest of the lakes [12]. The remaining three are mountain lakes located in the SE section of the MB national park, where deforestation and agricultural practices are less intense, and the lakes preserve clear, oligotrophic waters. Balamtetik, San Lorenzo, Bosque Azul and Peñasquito form a series of superficially interconnected plateau lakes, with Balamtetik directly receiving the inflow of the RGC. San Lorenzo and Bosque Azul are located further downstream while Peñasquito is a small doline next to Lake San Lorenzo, with a lower connectivity to the other plateau lakes. The rest of the MB lakes in this study are groundwater-fed and superficially isolated. The RGC basin includes the city of Comitán as well as other smaller settlements and areas of intensive agriculture. The higher turbidity and trophic conditions of the interconnected plateau lakes are considered to be a response to anthropogenic degradation processes affecting the MB region since 1986 [13] and more intensely since 2003 [14], when increasingly frequent reports of the local population pointed to changes in the colour and turbidity of these lakes.

### Sampling and analytical methods

#### Lake characterization.

The sixteen lakes were sampled during two fieldwork seasons. The first one was in July 2013 when seven lakes in the LF and five lakes in MB were sampled. The second was in November 2019, when the remaining four lakes in MB were sampled (Bosque Azul, Montebello, San Jose and Tziscao). Access to the lakes was facilitated by the local communities as well as by the staff from the “Comisión Nacional de Areas Naturales Protegidas” (CONANP), at the “Naha-Metzabok” Flora and Fauna Protection Area (Sonia Ñanez Jiménez, Miguel García Cruz) and the “Lagunas de Montebello” National Park (Edda Carolina Gonzalez del Castillo, Adolfo Vital Rumebe). In all cases, surface water samples (0.5 m) for total dissolved solids concentration (TDS) and major ion composition were collected from one location at a central part of each lake. Secchi disk depth (Z_SD_) was determined *in situ* and vertical profiles of pH, temperature, dissolved oxygen, and electrical conductivity were measured using a multiparametric probe (Hydrolab Quanta G in 2013 and Hydrolab DS5 in 2019). Samples for cation determinations were acidified with HNO_3_ and refrigerated until they were analyzed. Major ion determinations in 2013 were carried out with standard spectrophotometric methodologies [15] and in 2019 with ion chromatography using a Waters 717 Plus autosampler and a Waters 432 electrical conductivity detector. Ion concentrations expressed as mg L^-1^ were added to determine TDS. Ionic dominance was determined by transforming ion concentrations to meq L^-1^ and then to percentages (%Ca^2+^, %Mg^2+^, % [Na^+ ^+ % K^+^]; % [HCO_3_^- ^+ % CO_3_^2-^], %Cl^-^, %SO_4_^2-^). Water samples for chlorophyll *a* (Chla) and nutrient concentration analyses were also collected. Samples for Chla determinations were filtered (Whatman GF/C filters) and Chla was extracted with 90% methanol and measured spectrophotometrically (samples collected in 2013) or extracted with 90% acetone and measured by fluorescence (samples collected in 2019); concentrations were expressed as mg m^-3^. In 2013 the samples for ammonium and nitrates were acidified using H_2_SO_4._ Ammonium (N-NH_4_, Nessler’s method), nitrites (N-NO_2_, diazotization), nitrates (N-NO_3_, brucine colorimetric method), total phosphorus (TP, persulfate digestion), soluble reactive phosphorous (SRP, ascorbic acid method), and soluble reactive silica (SRSi, molybdate method) were determined in a Thermo Scientific GENESYS 20 visible spectrophotometer. Nutrient analyses for the four lakes sampled in 2019 were not possible, and determinations from a previous field season in spring 2017 were used. The Z_SD_ and Chla data from 2017 compared to 2019 allow us to consider that the trophic condition of these four lakes remained stable between 2017 and 2019. These samples were filtered through cellulose acetate syringe filters (0.22‐μm pore), collected in polypropylene containers, and stored frozen until analysis (within 48 hr of sampling). Analyses of N-NH_4_, N-NO_2_, N-NO_3_, phosphorus (TP and SRP), and soluble reactive silica (SRSi) used a segmented‐flow Autoanalyser (Skalar Sanplus System). Nutrients concentrations were expressed as µM. Dissolved inorganic nitrogen (DIN) corresponds to the sum of ammonium, nitrites, and nitrates concentrations. The trophic status of the lakes was determined based on Z_SD_, Chla, and TP (transformed to µg L^-1^) using Lamparelli´s trophic state index (TSI) according to formulas 1–4:


$$TSI_{\left(ZSD\right)}=10\left[6--\left(lnSD/ln2\right)\right]$$ (1)

$$TSI_{\left(Chla\right)}=10[6--(0\mathrm{.92}--0-34lnChla/ln2)]$$ (2)

$$TSI_{\left(TP\right)}=10\left[6--\left(1\mathrm{.77}-0\mathrm{.42}lnTP/ln2\right)\right]$$ (3)

$$TSI=\left[TSI_{\left(ZSD\right)}+TSI_{\left(Chla\right)}+TSI_{\left(TP\right)}\right]/3$$ (4)

#### Surface sediment samples.

For modern diatom analyses, surface sediment samples (top 1 cm) were collected using a gravity corer from the central part of each of the 16 studied lakes. Subsamples of 0.5 g of dry sediment were treated with HCl (10%) to eliminate carbonates and H_2_O_2_ (30%) to eliminate organic matter; if necessary, concentrated HNO_3_ was used to accelerate organic matter elimination. Permanent slides were prepared with 200 μl aliquots of final solution using Naphrax and analysed under an Olympus BX50 microscope with differential interference contrast at 1000x magnification. Diatom relative abundances were determined based on diatom counts of a minimum of 200 valves, except for Lake Balamtetik where only 100 valves were counted due to a low diatom valve concentration. Lake Amarillo (in the LF) was excluded from the diatom analysis because diatom valves were too scarce. Valve dissolution was observed in some of the planktonic taxa, mostly *Cyclotella petenensis*. Observations under the scanning electron microscope (JEOL JSM6360LV and JEOL NeoScope JCM-600) were undertaken to confirm the taxonomic identity of the most abundant diatom taxa. Diatom identification followed specialized literature [16–20].

#### Sediment sequence from Lake Peñasquito.

Lake Peñasquito is one of the currently eutrophic, turbid plateau lakes in MB. This lake was selected for paleolimnological analysis to determine the onset of eutrophication, and to establish its baseline condition prior to that change. For this purpose, a 73 cm long gravity core was recovered from the central part of this lake, at 43 m depth. This sequence was sampled every 1 cm, recording colour and texture of the sediments. Samples were freeze dried and homogenized using an agate mortar. The chronology of the sequence was established using the ^210^Pb-dating method and one accelerator mass spectrometry (AMS) radiocarbon age determination (Beta Analytic) at the bottom sample of the sequence (73 cm). The AMS radiocarbon age was calibrated using the CALIBomb program [21]. For the ^210^Pb dating, total ^210^Pb and ^226^Ra (supported ^210^Pb) were determined by high-resolution low-background gamma spectrometry (Ortec HPGe well detector) using ~2 mL of ground sediments stored in high-density plastic vials (closed with hermetic rubber stoppers and sealed with Teflon© tape) for 21 days, to ensure equilibrium between 226-Ra and Ra daughters [22]. Excess ^210^Pb (^210^Pb_exc_) was estimated as the difference between total and supported ^210^Pb. The age-depth model was constructed using PLUM [23]. Titanium (Ti) concentrations were determined in all the samples by energy dispersive X-ray fluorescence (ED-XRF) using a Thermo-Fisher Scientific Niton XL3t portable equipment. Titanium is a conservative, lithogenic element and its concentrations in lake sediments are related to erosion rates, increasing with a higher input of sediments from the basin [24–26]. For diatom analysis, a selection of non-homogenized samples spaced on average by 3 cm were prepared following the same methodology as for the surface sediment samples. Diatom counts for Lake Peñasquito were always above 300 valves. During diatom counts the number of chrysophyte cysts and scales were also recorded.

#### Modern diatoms data set analyses.

**Species composition.** The species with the largest regional occupancy in the modern diatoms data set were identified using a frequency of occurrence vs. mean relative abundance graph. Frequency of occurrence was determined as the percentage of the sites where each species was present and the mean relative abundance as the average of their relative abundances at the sites where they were present (sites with abundance zero were not considered). The species with the largest regional occupancy had a frequency of occurrence higher than 20% and a mean relative abundance above 5%. The Continental Algae Data Base [27] was used to verify if diatom species had been previously reported for Mexico. To assess the dispersal potential of the largest occupancy taxa, their ecological guilds were determined according to Benito et al. (2018).

**True diversity metrics**. To explore the diversity of the modern diatoms data set, the alpha, beta and gamma diversities were estimated using the true diversity metrics (^q^*D=*[Σp_i_^q^]^1/(1-q)^) of order q = 0, 1 and 2 [28–30]. The true diversity q = 0 is the species richness (^0^*D *= S) and represents the number of taxa present in each sample. The true diversity q = 1 is the Shannon diversity (^1^*D* = exp *H´,* where *H *= Shannon´s diversity index), representing the number of evenly distributed species in a sample. The true diversity q = 2 is the Simpson diversity (^2^*D *= 1/D, where D = Simpson´s diversity index) and represents the number of dominant species in a sample, which can fluctuate between 1 (highest dominance) and ^0^*D*. Alpha diversity is the average species richness in the samples (α = ^0^*D*_*avg*_*),* while gamma diversity is the species richness in the entire data set (γ = ^0^*D*_tot_). Sampling completeness for the diatom diversity estimations at each site (^0^*D*, ^1^*D* and ^2^*D*) was assessed using the sample coverage tests proposed by [31] using the R package “iNEXT” [32,33]

Beta diversity (β_w_ = γ/ α) [34] reflects the biological complexity of the region and represents the number of different communities in the studied area (metacommunity). Beta diversity is lower when one community dominates the landscape, so minimal species turnover between sampling units is expected, and it increases as the communities share a lower number of species in the landscape [29], whether this is related to species turnover (replacement) or nestedness (reduction in the number of species). The turnover (β_SIM_) and nestedness (β_SNE_) components of the beta diversity were estimated based on an absence/presence matrix and Sørensen dissimilarities, using the “betapart” package [35] in R (version 3.6.0) [32]. To determine whether there were significant differences in the species richness, Shannon and Simpson diversity metrics (q = 0, 1, 2) between lakes we utilized the 95% confidence intervals derived from the bootstrap method based on 500 replications in the iNEXT package in R [33]. If the confidence intervals for any two lakes did not overlap, we considered the differences to be statistically significant [30].

**Species distribution along environmental gradients**. To explore diatom species distributions along environmental gradients a canonical correspondence analysis (CCA) was performed [36]. Variance inflation factors (VIF) were determined and used to select variables, avoiding those with high correlation between them (VIF > 12). The eight selected variables included: water temperature, TDS, Chla, Z_SD_, DIN, SRP, %Ca^2+^ and %SO_4_^2-^. To improve the linearity and homogeneity of variances the diatom species relative abundances were transformed using square root and the environmental variables expressed as concentrations (SDT, Chla, DIN, and SRP) were transformed using logarithm (log_10_ + 1). The “downweight” function was used to reduce the influence of rare species and a series of individual CCAs were run to explore the importance of each variable at explaining diatom distribution. A Monte Carlo permutation test (999 permutations) was used to determine the statistical significance of each CCA. These analyses were performed using the “vegan” package (version 2.5.5) [37] in R (version3.6.0) [32].

## Results

### Characteristics of the studied lakes

**Fig 2:**
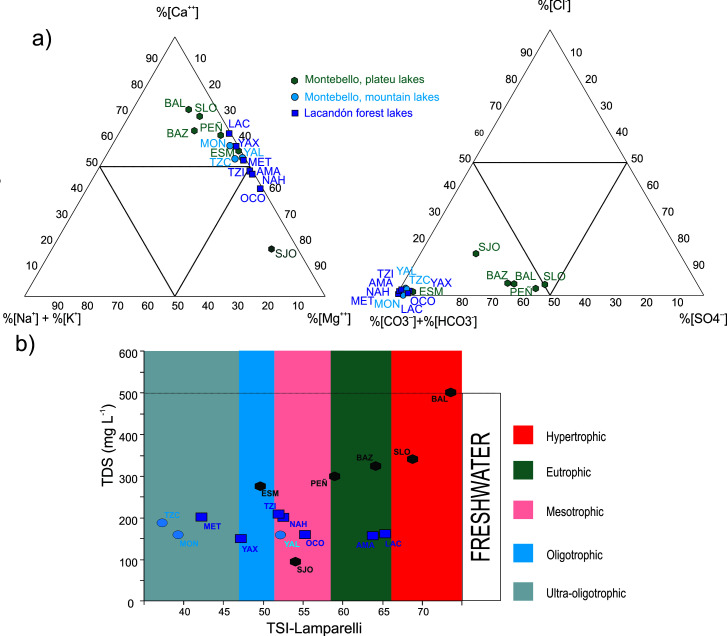
Ionic dominance, salinity (TDS) and Lamparelli´s trophic status index (TSI) of 16 Neotropical karstic lakes in southern México. 2a) Ionic dominance shows that most Montebello plateau lakes are separated by their higher sulphate proportions. 2b) Lamparelli´s trophic status index (TSI) compared to total dissolved solids (TDS), showing that four of the MB plateau lakes have a high trophic status (eutrophic to hypertrophic) and the highest salinities (300–500 mg L^-1^). The full names of the lakes and codes in Table 1.

All were alkaline (pH 7.4 to 9.2), freshwater lakes (TDS ≤ 500 mg L^-1^), dominated by %HCO_3_^-^ – %Ca^2+^ ~ %Mg^2+^ and ranging from ultra-oligotrophic to hypertrophic, according to Lamparelli´s TSI values (Fig 2, Table 1). However, the four interconnected plateau lakes in MB (Balamtetik, San Lorenzo, Bosque Azul and Peñasquito) stand out because they had higher %SO_4_^2-^ and ^-^%Cl^-^ (Fig 2a), slightly higher salinity (TDS ≥ 300 mg L^-1^) and high TSI values (Fig 2b).

All the lakes had DIN:TP below the critical 16:1 Redfield value [38], suggesting that at least seasonally nitrogen could be limiting the productivity of these lakes (Table 1). However, only five of the lakes (Esmeralda, Tziscao, Montebello, Metzabok and Yaxha) had DIN values below the phytoplankton starvation limit of 7 µM (Table 1), suggested by [39] and two (Tziscao and Montebello) had SRP values below the phytoplankton starvation limit of 0.1 µM. SRSi values were low (< 100 µM) in most lakes except for Balamtetik and San Lorenzo.

#### Species composition and diversity in the modern diatoms data set.

**Fig 3:**
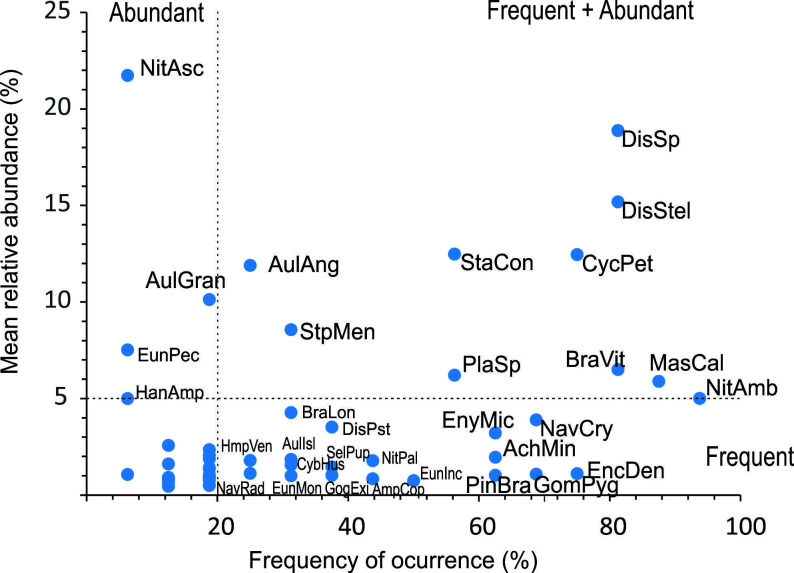
Regional occupancy diagram of the diatom taxa recorded in karstic lakes in southern Mexico. Frequent species were present in >20% of the lakes, abundant species had mean relative abundances ≥5%. Species full names, authorities and abbreviations are presented in S1 Table. Species abundances at each site are presented in S3 Fig.

**Fig 4:**
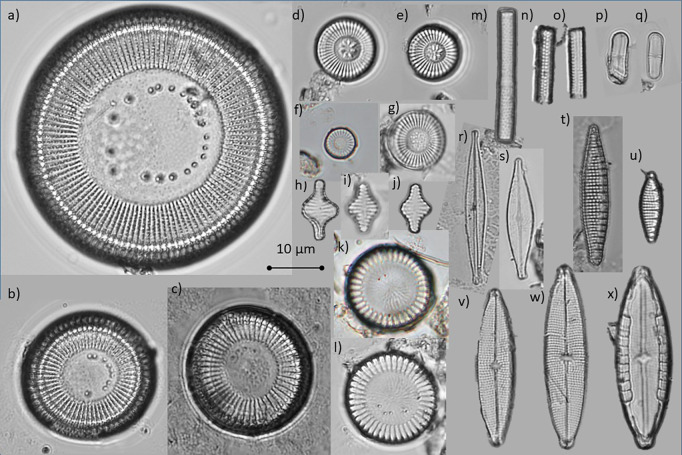
Plate showing the ten high regional occupancy diatom taxa in the studied karstic lakes from southern Mexico. 4a-4c) *Cyclotella petenensis,* 4d - 4e) *Discostella stelligera,* 4f - 4g) *Discostella* sp.*,* 4h - 4j) *Staurosira construens,* 4k - 4l) *Stephanocyclus meneghinianus,* 4m - 4o) *Aulacoseira granulata* var*. angustissima,* 4p - 4q) *Planothidium* sp.*,* 4r - 4s) *Brachysira vitrea,* 4t - 4u) *Nitzschia amphibioides,* 4v - 4x) *Mastogloia calcarea.*

An average of 370 valves per sample was counted in the 15 lakes included in the diversity and statistical analyses (S1 Table). Lake Amarillo was excluded as diatom counts were too low. Diatom counts in these 15 lakes ranged from 100 valves in Balamtetik to 710 in Yalalush. The sample coverage tests (S1 Table) allowed to establish that these counts documented on average 79% of its species richness (^0^*D*), 95% of its Shannon diversity (^1^*D*) and 98% of its Simpson diversity (^2^*D*). While Shannon and Simpson diversities were well documented in all the lakes (≥90%), species richness values were somewhat low (< 80%) in four lakes in the LF and two in MB, where only about 50–75% of the species richness was documented. Therefore the 50 diatom taxa identified (γ diversity) represent a minimum estimate of the species richness in the region (S2 Table). According to the Continental Algae Database [27] four of these taxa (8%) represented first reports for Mexico. Six (1.2%) could not be assigned to any described species and might represent new, undescribed species. Ten of the 50 diatom taxa identified (20%) had a high regional occupancy (frequencies of occurrence > 20% and relative abundances ≥ 5%, Fig 3). These were: *Aulacoseira granulata* var*. angustissima*, *Brachysira vitrea, Cyclotella petenensis, Discostella stelligera, Discostella* sp., *Mastogloia calcarea, Nitzschia amphibioides, Planothidium* sp*., Staurosira construens,* and *Stephanocyclus meneghinianus* (Fig 4). According to [40] all of them, except for *B. vitrea* and *Planothidium* sp., have high dispersal potentials as they are either planktonic or free-motile taxa. *B. vitrea* and *Planothidium* sp. have a lower dispersal potential as they are attached-low profile species.

**Fig 5:**
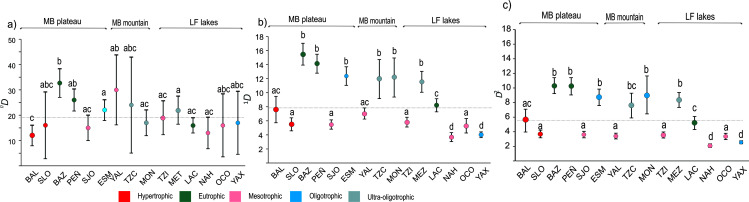
True diversity metrics for the studied karstic lakes in southern Mexico. 5a - 5c) True diversity metrics of order q = 0, 1 and 2, with 95% confidence intervals. Dotted lines denote average values. Letters denote statistically significant groups.

The species richness per site (^0^*D*) ranged from 12 to 32 taxa, with an average (α diversity) of 19 species (α _=_ 19.3), even though these numbers are only minimum estimates. Nevertheless, when the 95% confidence intervals were considered, the values of all the lakes overlapped, showing no significant differences in species richness among them (Fig 5a). Average Shannon diversity (^1^*D*) was of nearly eight effective species per site (^1^*D*_*avg*_ = 8.2, range 3.7 to 15.4). In this case, when the 95% confidence intervals were considered, six lakes (Bosque Azul, Peñasquito, Esmeralda, Tziscao, Montebello and Metzabok) were identified by a higher Shannon diversity (^1^*D* > 10) (Fig 5b). Regarding Simpson diversity (^2^*D*), the average was of nearly six co-dominant species per site (^2^*D*_*avg*_ = 5.5, range from 2.1 to 10.2) (Fig 5c) and when the 95% confidence intervals were considered, a group of seven lakes (San Lorenzo, San Jose, Yalalush, Tzi´Bana, Naha, and Ocotalito) with a higher dominance (lower number of co-dominant taxa, ^2^*D* < 4) could be identified. However, it was not clear which lake attributes could be responsible for the higher or lower diversity values (^1^*D* or ^2^*D*) in the lake groups, with no apparent correlation with trophic status or lake salinity. At a regional scale, the beta diversity was estimated to be between 2 and 3 effective assemblages in the area (β_w_ = 2.6), with a high turnover (β_SIM_ = 0.83) and a small nestedness component (β_SNE _= 0.09).

### Modern diatom species distribution along environmental gradients

**Fig 6:**
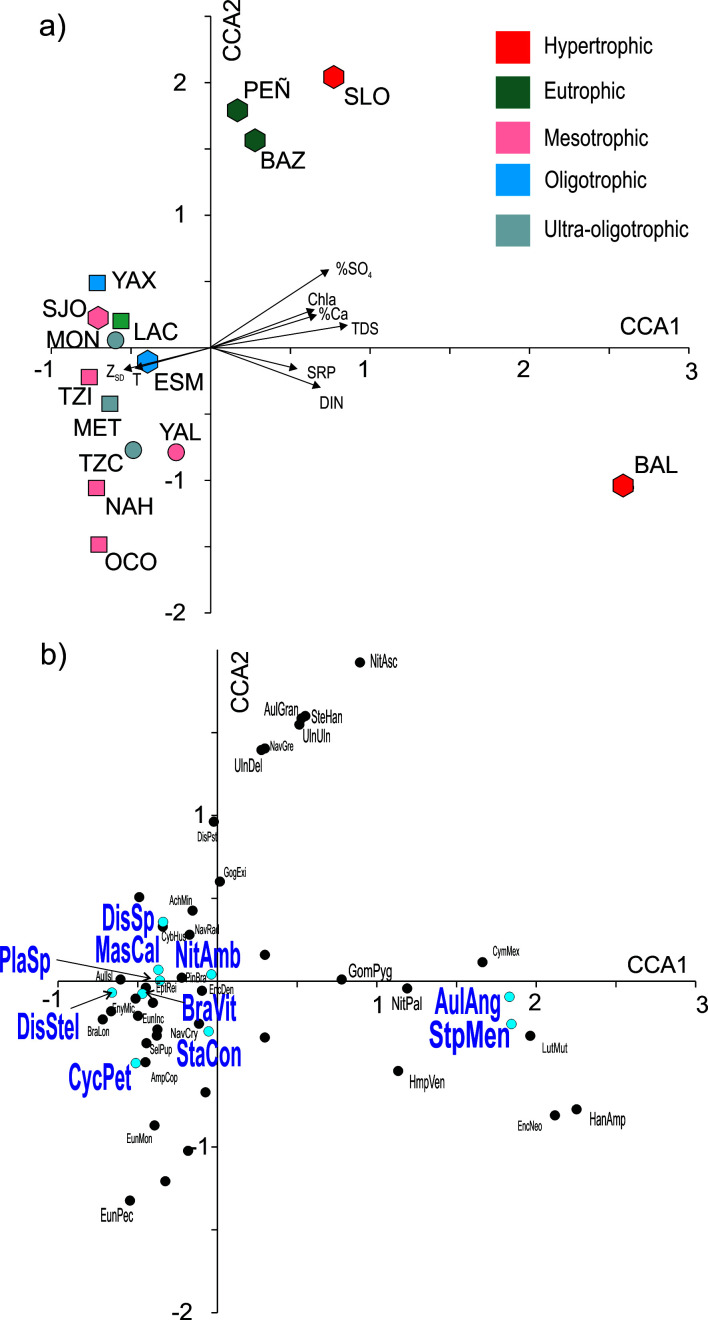
Canonical correspondence analysis (CCA, axis 1 vs. axis 2) for lake attributes and diatom species relative abundances for 15 Neotropical karst lakes in southern Mexico. 6a) Sites plot, with trophic classification according to Lamparelli´s trophic state index. 6b) Species plot. TDS = Total dissolved solids, T = water temperature, SRP = soluble reactive phosphorous, Z_SD_ = Secchi disk depth, DIN = dissolved inorganic nitrogen. Sites abbreviations and full names as in Table 1. Species full names and codes are in S1 Table.

The CCA model was significant (p < 0.005), and the VIF of all variables were low (VIF < 12), indicating a low correlation between them (Fig 6). The first two axes explained 40.3% of the data variation. The individual CCAs showed that the variables with the highest significance in explaining diatom distribution in the data set were TDS and %SO_4_^2-^ (p < 0.001), followed by Chla (p < 0.005) and with a lower significance also DIN (p < 0.05). Axis 1 (λ = 0.57, p = 0.005, proportion explained = 30.3%) correlated positively with these four variables. None of the eight attributes of the lakes showed a high correlation with axis 2. In the axis 1 vs. axis 2 plot, two main groups of lakes could be identified, on the positive side of axis 1 were the interconnected plateau lakes in MB, with high TDS, %SO_4_^2-^, Chla and DIN: Balamtetik, San Lorenzo, Bosque Azul and Peñasquito. Amongst these, Lake Balamtetik had the highest axis 1 scores showing that it is the most impacted lake in the data set, as it is the one that directly receives the inflow of the RGC. The diatom species that were characteristic of this group of lakes (positive scores on axis 1) included two of the high regional occupancy taxa, *Stephanocyclus meneghinianus* and *Aulacoseira granulata* var*. angustissima,* these diatoms were absent in the rest of the lakes. Other taxa with positive axis 1 scores were: *A. granulata, Gomphonema pygmaeum*, *Halamphora veneta*, *Hantzschia amphioxys*, *Nitzschia palea*, *N. ascicularis*, *Stephanodiscus hantzschii* and *Ulnaria delicatissima*. The high position along axis 1 of Lake Balamtetik shows a complete species turnover in this lake with respect to those on the negative side of axis 1. In contrast San Lorenzo, Bosque Azul and Peñasquito had an intermediate position, reflecting a partial species turnover. The lakes with negative axis 1 scores had lower TDS, %SO_4_^2-^, Chla and DIN values, and included the non-superficially interconnected lakes in MB as well as all the lakes in the LF. The diatoms on the negative side of axis 1 included the remaining eight of the ten high regional occupancy taxa: *Cyclotella petenensis, Brachysira vitrea, Discostella* sp., *Discostella stelligera, Mastogloia calcarea, Nitzschia amphibioides, Planothidium* sp., and *Staurosira construens*. The lakes and species in this group (negative axis 1 scores) reflect a partial species turnover along axis 2. At the negative end of this axis were two lakes dominated by the large planktonic species *C. petenensis* (lakes Naha and Ocotalito, in the LF). These were followed by two lakes where tychoplanktonic *S. construens* was most abundant (lakes Tziscao and Yalalush in MB). At the higher axis 2 scores were the lakes where the smaller planktonic *D. stelligera* and *Discostella* sp. had the highest abundances (lakes Yaxha, San José, and Montebello).

### The paleolimnological record from Lake Peñasquito

**Fig 7:**
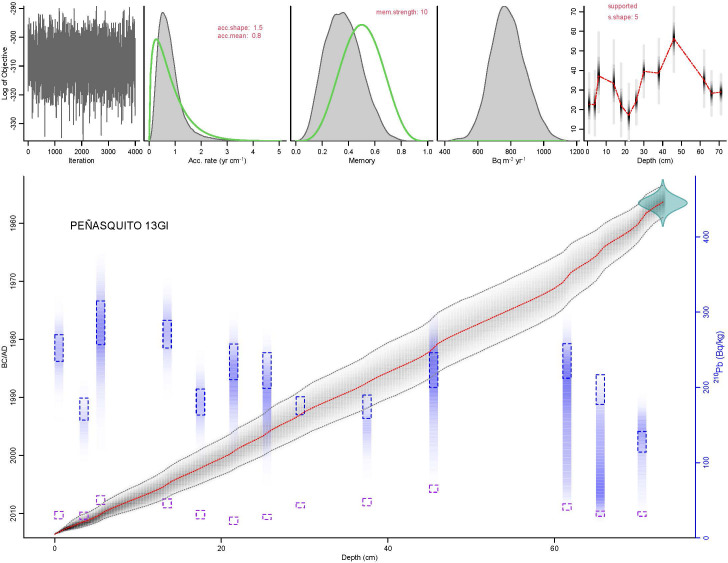
Age-depth model from Lake Peñasquito, Montebello lakes region, southern Mexico. The model was constructed using the program PLUM, based on ^210^Pb determinations and one radiocarbon date for the 73 cm long sediment sequence recovered.

**Fig 8:**
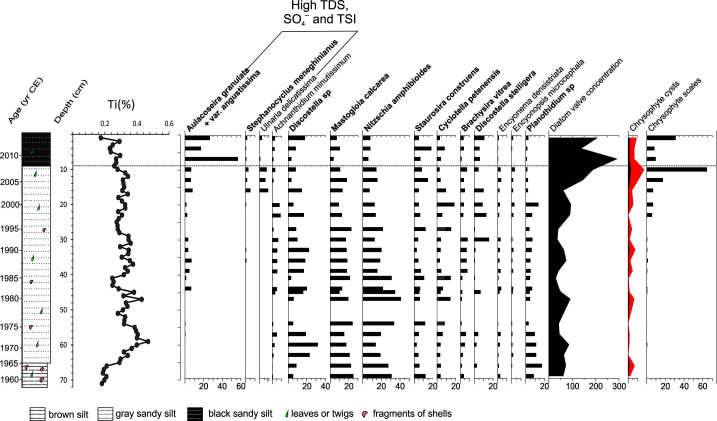
Paleolimnological record from Lake Peñasquito, Montebello lakes region, southern Mexico. The figure includes the sediment stratigraphy, titanium concentration (Ti %), main diatom species (%), and diatom valves, Chrysopyte cysts and scales concentrations per gram of dry sediment (gds).

The AMS radiocarbon age at the base of the Peñasquito sediment sequence (Beta− 376718, 73 cm) was 104.3 ± 0.3% postmodern carbon (pMC) which after calibration gave a calendar age of 1956–1957 yr CE. This date together with the ^210^Pb results allowed us to establish an age-depth model (Fig 7) based on which it was estimated that the average time resolution of the samples spaced every 1 cm was of about 0.8 years and of the diatom samples spaced every 3 cm was of about 2.4 years. The sediments along the core (Fig 8) were brown silts (73–65 cm, ~ 1956–1966), that changed to gray sandy silts (65–9 cm, 1966–2008) and to black sandy silts on the top (9–0 cm, 2008–2013). The bottom sediments had the lowest Ti values (< 0.22%) that sharply increased in the gray sandy silts (Fig 8). The highest Ti values (0.47%) were reached at 59 cm (~1972 ± 5 CE), with further peaks at 47–45 cm (~1981 ± 5) and from 39–30 cm (~1986–1993).

The diatom assemblage in the bottom sediments and up to 45 cm depth (~1982 ± 5) included the eight high regional occupancy diatom taxa with negative axis 1 scores in the modern diatoms CCA. According to the CCA results, these species indicate low salinity, low sulphates and low Chla values. The most abundant were *Discostella* sp., *Mastogloia calcarea, Nitzschia amphibioides*, and *Planothidium* sp., but also present were *Staurosira construens, Cyclotella petenensis,* and *Discostella stelligera*. The Ti peaks at 47–45 cm (~1981 ± 5) were followed by a change in the diatom assemblage, which now included low abundances (< 10%) of *A. granulata* and its var. *angustissima.* This diatom assemblage remained stable until 16 cm depth (~2003 ± 3), when low abundances (<10%) of *Stephanodiscus meneghinianus* and *Ulnaria delicatissima* were also incorporated. These taxa (*A. granulata* + var. *angustissima*, *S. meneghinianus* and *U. delicatissima*) are part of the diatom species group with positive axis 1 scores on the modern diatoms CCA, with an affinity for higher lake water salinity, sulphates, and Chla values. On the other hand, many of the initially abundant taxa from the negative axis 1 scores group in the modern diatoms CCA showed a gradual decrease. Total diatom abundance as well as chrysophyte scales and cysts concentrations sharply increased from 16 to 9 cm depth (~2003–2008). The top black sediments (~2008–2013) showed the highest diatom abundances and percentages of *A. granulata* + var. *angustissima* (up to 60%).

## Discussion

### Modern diatom species composition and diversity in Neotropical karst lakes in southern Mexico

This work is the first one reporting a detailed survey of the diatom species diversity and ecological distributions in the Neotropical mountain-karst region in southern Mexico. We documented 50 species present in these lakes, with ten having a high regional occupancy. This is a minimum estimate that represents about 80% of the diatom species richness (γ diversity) of the region. This number falls withing the diversity range of similar surveys in central Mexico [41,42] and the Yucatán-Guatemala region [18,43], considering that this one included a lower number of sites. There was no correlation between species diversity and environmental factors such as salinity or trophic conditions of the lakes. Nevertheless, species distribution between the lakes responded primarily to their hydrochemical characteristics (TDS and %SO_4_) and trophic condition (Chla and DIN). The CCA analysis (axis 1 scores) identified that eight of the ten high regional occupancy taxa were characteristic of lower-salinity and lower %SO_4,_ Chla and DIN concentrations; these species are representative of the relatively healthy ecosystems in the region (*Cyclotella petenensis, Brachysira vitrea, Discostella* sp., *Discostella stelligera, Mastogloia calcarea, Nitzschia amphibioides, Planothidium* sp., and *Staurosira construens*). On the other hand, the remaining two (*Aulacoseira granulata* var. *angustissima*, and *Stephanocyclus meneghinianus*) are indicators of human-induced hydrochemical changes in the lakes (higher salinity, %SO_4,_ Chla and DIN). The high species turnover identified in the beta diversity (β_sim_ = 0.83) is attributed to the total replacement of species in Lake Balamtetik (hypertrophic). The rest of the lakes, with lower human-impact showed a partial replacement of species. Furthermore, a transition in the dominant taxa of the non-impacted lakes was identified, from large, planktonic *Cyclotella petenensis* to smaller size *Discostella* spp. None of the analysed environmental variables could be associated with this replacement gradient, therefore we assume that it responds to factors that were not measured in this survey. Nevertheless, proliferation of small Cyclotelloid species such as *Discostella* spp. has been identified as a response to warmer conditions and enhanced water column stratification linked to global warming [44,45]. This is a likely explanation that is supported also by the paleolimnological record from Lake Montebello. Lake Montebello is one of the low human-impact, mountain lakes in the MB region in this data set. In this lake a change in diatom dominance was recorded around 2010, from large *C. petenensis* to small *Discostella* spp. This transition occurred as a response the increasingly warmer modern climatic conditions, after a high turbidity crisis in the lake that was caused by land use change and increasing erosion to the lake [46].

The relatively low β_w_ in the diatoms (β_w_ = 2.6) contrast with the high regional complexity found in the MB lakes for zooplankton (β_w _~ 6) and for benthic macroinvertebrates (β_w_ ~ 10) [47–50]. For these organisms nearly each lake had a distinctive species assemblage, and no significant correlations could be established between species distributions and environmental variables. This was not the case for diatom species where an environmental filter associated with human impact was identified as the main driver of species distribution. However, the partial turnover of species in the lower human-impact lakes follows a pattern that could be a response of yet unidentified factors, possibly associated with the local history of human-impact due to land use changes or to the impact of global environmental drivers such as global warming. This would be a similar case as was documented for the Orinoco River basin, where diatom distribution was largely controlled by a mixture of environmental filtering vs. historical legacies [51].

#### Observations on the geographic distributions of the high regional occupancy taxa.

The ten high regional occupancy species included three cosmopolitan planktonic taxa (*Discostella stelligera*, *Aulacoseira granulata* var. *angustissima* and *Stephanocyclus meneghinianus*) of high dispersal potential. These species were also important (frequent and abundant) in a survey (n = 46 sites) undertaken on non-karstic lakes in central Mexico [42]. Furthermore, these species have also been reported in diatom studies from different regions of the world, including the USA, South America and Africa [52–61]. These species show consistent ecological distributions at a broad regional level, and therefore environmental filtering seems to be the most important factor explaining the local distributions of these cosmopolitan taxa. In central Mexico the distribution of these species in the survey undertaken by [42] followed a salinity gradient, with *D. stelligera* at the lower end (TDS < 200 mg L^-1^), *A. granulata* var. *angustissima* in the middle (TDS = 200–500 mg L^-1^) and *S. meneghinianus* preferring higher salinities (TDS > 500 mg L^-1^). *A. granulata* var. *angustissima* and *S. meneghinianus* were also present in lakes with a high trophic status (eutrophic). *S. meneghinianus* (*= Cyclotella meneghiniana*) was also a high-frequency taxa in a survey undertaken in the Yucatán-Guatemala region [43] where it also showed an affinity for high trophic status environments, such as Lake Amatitlán. These ecological distributions agree with our findings for the karstic lakes in southern Mexico, as *D. stelligera* was common in the lower-salinity, oligo-mesotrophic lakes while *A. granulata* var. *angustissima* and *S. meneghinianus* were characteristic of the higher-salinity, eutrophic to hypertrophic plateau lakes in MB.

Contrastingly, the rest of the high occupancy taxa identified in the Neotropical karstic lakes in southern Mexico are absent or rare in the central-Mexico data set studied by [42]. Furthermore, despite having high dispersal potentials, some elements of this Neotropical-karst diatom flora showed restricted regional distributions. These taxa include the unidentified *Discostella* sp. and *Planothidium* sp., which could represent new species and *Mastogloia calcarea*, a relatively recently described taxon that very likely was previously misidentified in the region with *M. smithii* or *M. lacustris* (= *M. smithii* var. *lacustris*) [17]. So far, this last species has only been reported from the tropical karst region of the Caribbean Sea as well as the Florida and Yucatán peninsulas, and very likely it corresponds with reports of *M. smithii* in southern Mexico and Guatemala [17,18,26,43,62]. The present research extends its distribution to lakes in the Neotropical mountain-karst region in southern Mexico. Finally, *Cyclotella petenensis* is a species that was described from late Pleistocene fossil material from Lake Peten Itza, in the lowlands of Guatemala [19], but that has only been reported in low abundances in the modern environments of that region. The higher abundance of *C. petenensis* in the higher altitude lakes in this study gives a wider perspective of its ecological preferences, which are valuable for paleoenvironmental reconstructions. This species is part of the low-salinity, oligo-mesotrophic assemblage, and attained its highest abundances (~40%) in two of the LF lakes (Naha and Ocotalito), in relatively deep (>10 m), mesotrophic, slightly alkaline (pH = 7.7), low salinity (TDS < 200 µg/L, EC ≤ 200 µS cm^-1^) environments. The presence of this species in the Lake Petén Itza record provides an excellent example of how environmental conditions and historical factors can influence modern species distributions. During the last glacial maximum this species was abundant in the Guatemala lowlands, but it became rare or disappeared from that area at the late Pleistocene to Holocene climatic transition [19,63] but our results show that now it is abundant at higher altitude lakes, in the LF and MB regions. Comparable changes in species distribution in central Mexico during glacial/interglacial cycles were reported for *Stephanodicus niagarae* [64], as climate changes modified the geographic distribution where this species could be dispersed. The results of the present study also suggest that for *C. petenensis*, eutrophication and warming conditions represent stressor factors that might be restricting its distribution in the studied area while other taxa are favoured.

### History of lake disturbance

The realization that human induced changes in the Neotropical karstic region in southern Mexico were modifying these aquatic ecosystems dates from nearly three decades ago [13]. These authors expressed concern regarding the impact of wastewater inflow and agricultural lixiviates on the lakes through the RGC, indicating an already evident deterioration of the interconnected plateau Lakes Balamtetik, San Lorenzo and Bosque Azul (addressed as Tepancoapan system). More recent studies based on Chla values of 18 lakes in MB confirmed that trophic conditions of the interconnected lakes (such as Balamtetik and San Lorenzo) was higher (meso-eutrophic) than in the groundwater-fed lakes [12]. The results of the present study show that besides high trophic levels, there are other important changes in the hydrochemistry of the interconnected plateau lakes, which include higher salinities (TDS 300–500 mg L^-1^) and higher proportions of sulphates and chlorides (%SO_4_^2-^ and %Cl^-^). High trophic levels and hydrochemical changes can be attributed to urban sewage input and agricultural solutes derived from the use of sulphate-rich fertilizers as well as to soil-derived sediment and organic matter entering through the RGC [65–67]. Lake Balamtetik directly receives the inflow of the RGC and shows the strongest changes (highest TDS and TSI values) compared to the subsequent lakes in the chain (San Lorenzo, Bosque Azul). The modern diatom analysis performed in this study also showed that this lake is the one with a complete diatom species turnover compared to the rest of the lakes in the region.

Our results also showed that two diatom species can be identified as the main indicators of human-induced eutrophication and hydrochemical changes in the region, *Aulacoseira granulata* var. *angustissima* and *Stephanocyclus meneghinianus*, These two taxa were associated to a group of less abundant taxa which included *A. granulata, Gomphonema pygmaeum*, *Halamphora veneta*, *Hantzschia amphioxys*, *Nitzschia palea*, *N. ascicularis*, *Stephanodiscus hantzschii* and *Ulnaria delicatissima*. With this information, there are questions that we can address from a paleolimnological approach. For example, did the currently impacted lakes evolved from a relatively pristine condition as suggested by [14]? Which was the baseline condition for these lakes? How and when did the deterioration process occurred? None of these questions could be clearly addressed by our previous paleolimnological work on Lake Balamtetik [66] due to a poor chronological control, or on Lake San Lorenzo [26], because disturbance taxa (*A. granulata* var. *angustissima* and *S. meneghinianus*) were present along the whole studied sequences, which dated to ~1956. However, the solid age-depth model developed for Lake Peñasquito, provides good chronological constrain for the reconstruction of past events. The paleolimnological record from this lake shows a transition from a baseline condition to its currently eutrophic status. Furthermore, increases in Ti predate the first appearance and gradual increase in abundance of the diatom species identified as indicators of human induced hydrological and trophic level changes.

The sedimentary sequence from Peñasquito shows low erosion rates over the lake basin before 1965, when the lake had a diatom assemblage dominated by the eight high regional distribution taxa common to the lower-salinity, oligo-mesotrophic lakes. However, increasingly higher erosion rates affected the lake from ~1965 to ~1972 and during the 1980s. During this last decade is when [13] identified the first warning signals in the lakes. Increased erosion in a lake basin is a sign of land use changes as the agricultural horizon expanded, and human occupation increased. For example, at one of the municipalities in MB (La Trinitaria), the population increased 1.6 times between 1980 and 1990 and duplicated between 1980 and 2000 [66,68]. The sharp increase in erosion rates was shortly followed (~ 1982) by the incorporation in the diatom assemblage of *A. granulata* + var. *angustissima.* Approximately 20 years later (~2003 to ~2008), other indicators of hydrological changes and increased trophic conditions were also recorded, which included higher diatom and chysophyte productivity, besides the increase in *S. menghinianus* and *U. delicatissima* in the diatom assemblage. By ~2008 the lake crossed a critical point, reaching its current eutrophic condition characterised by the highest diatom productivity and abundances of *A. granulata* + var. *angustissima*. However, Lake Peñasquito still seems to be in a transitional phase, which could culminate with a total extirpation of its original diatom diversity, as has happened in Lake Balamtetik. We must bear in mind that each lake has an “individual” story determined by its interconnectivity and historical legacy, and that Lake Peñasquito, while superficially interconnected to the other plateau lakes, is not part of the main lake chain receiving the inflow of the RGC, therefore its deterioration process was somewhat slower with respect to those lakes directly in line with the RGC discharge, such as Balamtetik, San Lorenzo and Bosque Azul. Nevertheless, the history of this lake is representative of the degradation processes occurring in the Neotropical karstic region in southern Mexico during the last century, showing a long history of disturbance that began since the 1960s and that has increasingly affected the lakes. It is also a warning story showing that sooner or later, other lakes in the karstic system could reach a deterioration braking point.

## Conclusions

1. Our results support that soil-derived sediment and organic matter, urban sewage and agricultural solutes originating from sulphate-rich fertilizers enter the MB plateau lakes through the RGC, causing higher salinity (TDS 300–500 mg L^-1^), higher %SO_4_^2-^ and eutrophic to hypertrophic conditions in the interconnected lakes: Balamtetik, San Lorenzo, Bosque Azul and Peñasquito.
2. We identified two ecological groups in the diatom species, driven mainly by ionic concentration, ionic composition and trophic level. This separation explained most of the regional complexity and high species turnover identified by the beta diversity (β_w_ = 2.6, β_SIM_ = 0.83).
3. The first ecological group included two high regional occupancy taxa (*Aulacoseira granulata* var. *angustissma* and *S. meneghinianus)* indicative of the higher lake salinity (TDS), % SO_4_^2-^, Chla and DIN that were abundant in the diatom assemblages of four highly impacted and interconnected plateau lakes in MB.
4. The second ecological group included eight high regional occupancy taxa, characteristic of the lower human-impacted lakes (low TDS, %SO_4_^2-^, Chla and DIN: *Cyclotella petenensis, Brachysira vitrea, Discostella* sp., *D. stelligera, Mastogloia calcarea, Nitzschia amphibioides, Planothidium* sp., and *Staurosira construens).* Within this second group of lakes, a partial species turnover was identified, from those dominated by large planktonic *Cylcotella petenensis* to those dominated by smaller planktonic *Discostella* spp. It is possible that this trend is associated with historical factors such as the local history of human disturbance at each lake as well as to the impact of modern global warming.
5. The ten high regional occupancy taxa were mainly species with a high dispersal potential (planktonic or free-motile). Three of these species (*D. stelligera*, *A. granulata* var. *angustissima*, and *S. meneghinianus)* are cosmopolitan taxa that show consistent ecological distributions at a broad regional level. Their local abundances are controlled mostly by environmental filtering, and their distribution follows a salinity gradient.
6. At least four of the high regional occupancy taxa have a restricted distribution in the Neotropical karst region: *Mastogloia calcarea*, *Cyclotella petenensis*, *Discostella* sp. and *Planothidium* sp. The distribution of these species is not constrained by dispersal limitations but by environmental filtering and historical factors such as climatic changes. These species could be at risk of extirpation from their natural habitats in the scenario of increasing environmental change in the region.
7. The record from Lake Peñasquito shows a gradual transition, from a baseline condition prior to ~1965 with a diatom assemblage characteristic of low salinity and trophic level, passing through an initial degradation period during the 1980s to an accelerated degradation process from ~2003–2008, when the lake reached its current eutrophic condition, and the diatom assemblage became dominated by one cosmopolitan taxon. The history of this lake is representative of the degradation process occurring in these Neotropical karstic lakes under increasing human impact.
8. Conservation policies in this region should prioritize the protection of lakes critical to maintaining regional diatom diversity, as these ecosystems support unique assemblages that reflect current environmental conditions and long-term ecological processes.

## Supporting information

S1 Table Valve counts and sample coverage test for species richness (^0^*D*), Shannon (^1^*D*) and Simpson (^2^*D*) diversity. (DOCX)

S2 Table Taxonomic list of the diatom taxa found in the studied lakes in Chiapas. (DOCX)

S3 Fig Relative abundances of the diatom taxa present in the studied karstic lakes in southern Mexico. White bars in *Cyclotella petenensis* represent the percentage of valves showing dissolution. LF = Lacandona region lakes, MBm = Montebello region mountain lakes, MBp = Montebello region plateau lakes.
(DOCX)
