# Supplementary material for: Diatom diversity and distribution in Neotropical karst lakes under anthropogenic stress
Source: PLoS One. 2025 Jul 24;20(7):e0327201. doi: 10.1371/journal.pone.0327201 (PMC12289067; doi:10.1371/journal.pone.0327201)
Supplement: S1 Table — (DOCX) [file pone.0327201.s001.docx]

**S1 Table. Valve counts and sample coverage test for species richness (^0^*D*), Shannon (^1^*D*) and Simpson (^2^*D*) diversity.**

|  |  |  | sample completness (%) | | |
| --- | --- | --- | --- | --- | --- |
|  | Lake | Valves counted | ^0^*D* | ^1^*D* | ^2^*D* |
| LF | Amarillo* | 28 | 44 | 78 | 97 |
|  | Nahá | 287 | 74 | 97 | 100 |
|  | Yaxha | 675 | 58 | 98 | 100 |
|  | Ocotalito | 226 | 47 | 93 | 99 |
|  | Metzabok | 326 | 83 | 96 | 98 |
|  | Tzibaná | 490 | 66 | 97 | 99 |
|  | Lacandón | 333 | 98 | 98 | 99 |
| LF average* |  | 390 | 71 | 96 | 99 |
|  |  |  |  |  |  |
| MB | Balamtetik | 100 | 86 | 92 | 94 |
|  | SanLorenzo | 253 | 56 | 95 | 99 |
|  | San José | 310 | 99 | 98 | 99 |
|  | Esmeralda | 415 | 99 | 97 | 98 |
|  | BosqueAzul | 466 | 89 | 96 | 98 |
|  | Peñasquito | 577 | 98 | 98 | 98 |
|  | Montebello | 202 | 97 | 90 | 90 |
|  | Tziscao | 200 | 50 | 89 | 96 |
|  | Yalalush | 710 | 83 | 97 | 100 |
| MB average |  | 359 | 84 | 95 | 97 |
|  |  |  |  |  |  |
| Global average* |  | 371 | 79 | 95 | 98 |
|  |  |  |  |  |  |
|  |  |  | * Lake Amarillo was excluded | | |

LF= Lacandon Forest region, MB = Montebello Lakes region.
