# Supplementary material for: Diatom diversity and distribution in Neotropical karst lakes under anthropogenic stress
Source: PLoS One. 2025 Jul 24;20(7):e0327201. doi: 10.1371/journal.pone.0327201 (PMC12289067; doi:10.1371/journal.pone.0327201)
Supplement: S2 Table — (DOCX) [file pone.0327201.s002.docx]

**S2 Table. Taxonomic list of the diatom taxa found in the studied lakes in Chiapas.**

| **Coscinodiscophyceae** | |  |  | **Code** |  |
| --- | --- | --- | --- | --- | --- |
|  | Aulacoseirales | |  |  |  |
|  |  | Aulacoseiraceae | |  |  |
|  |  |  | *Aulacoseira granulata* (Eherenberg) Simonsen 1979 | AulGra |  |
|  |  |  | *Aulacoseira granulata* var. *angustissima* (Müller) Simonsen 1979 | AulAng |  |
|  |  |  | *Aulacoseira* aff*. islandica* (O.Müller) Simonsen 1979 | AulIsl |  |
| **Mediophyceae** | |  |  |  |  |
|  | Stephanodiscales | |  |  |  |
|  |  | Stephanodiscaceae | |  |  |
|  |  |  | *Cyclotella petenensis* Sylvestre, Paillès & Escobar 2018 | CycPet | ** |
|  |  |  | *Discostella pseudostelligera* (Hustedt) Houk & Klee 2004 | DisPse |  |
|  |  |  | *Discostella stelligera* (Cleve & Grunow) Houk & Klee 2004 | DisSte |  |
|  |  |  | *Discostella* sp*.* |  |  |
|  |  |  | *Stephanocyclus meneghinianus* (Kützing) Kulikovskiy, Genkal & Kociolek 2022 | SteMen |  |
|  |  |  | *Stephanodiscus hantzschii* Grunow 1880 | SteHan |  |
| **Bacillariophyceae** | |  |  |  |  |
| **Fragilariophycidae** | |  |  |  |  |
|  | Fragilariales |  |  |  |  |
|  |  | Staurosiraceae | |  |  |
|  |  |  | *Staurosira construens* Ehrenberg 1843 | StaCon |  |
|  |  |  | *Staurosira venter* (Ehrenberg) Cleve & Möller 1879 | StaCnv |  |
|  | Licmophorales | |  |  |  |
|  |  | Ulnariaceae |  |  |  |
|  |  |  | *Ulnaria delicatissima* (W.Smith) Aboal & P.C.Silva 2004 | UlnDeli |  |
|  |  |  | *Ulnaria ulna* (Nitzsch) Compère 2001 | UlnUln |  |
| **Eunotiophycidae** | |  |  |  |  |
|  | Eunotiales |  |  |  |  |
|  |  | Eunotiaceae |  |  |  |
|  |  |  | *Eunotia incisa* Smith ex Gregory 1854 | EunInc |  |
|  |  |  | *Eunotia monodon* Ehrenberg 1843 | EunMon |  |
|  |  |  | *Eunotia pectinalis* (Kützing) Rabenhorst 1864 | EunPec |  |
| **Bacillariophycidae** | |  |  |  |  |
|  | Achnanthales | |  |  |  |
|  |  | Achnanthidiaceae | |  |  |
|  |  |  | *Achnanthidium minutissimum* (Kützing) Czarnecki 1994 | AchMin |  |
|  |  |  | *Gogorevia exilis* (Kützing) Kulikovskiy & Kociolek 2020 | GogExi |  |
|  |  |  | *Planothidium* sp. | PlaSp |  |
|  |  |  |  |  |  |
|  |  |  |  |  |  |
|  | Naviculales |  |  |  |  |
|  |  | Amphipleuraceae | |  |  |
|  |  |  | *Halamphora veneta* (Kützing) Levkov 2009 | HmpVen |  |
|  |  | Brachysiraceae | |  |  |
|  |  |  | *Brachysira longirostris* (Hustedt) D.G.Mann 1990 | BraLon | ** |
|  |  |  | *Brachysira vitrea* (Grunow) Ross 1966 | BraVit |  |
|  |  |  | *Nupela* sp. | NupSp |  |
|  |  | Diadesmidaceae | |  |  |
|  |  |  | *Luticola mutica* (Kützing) Mann 1990 | LutMut |  |
|  |  | Diploneidaceae | |  |  |
|  |  |  | *Diploneis ovalis* (Hilse) Cleve 1891 | DipOva |  |
|  |  | Naviculaceae |  |  |  |
|  |  |  | *Navicula cryptocephala* Kützing 1844 | NavCry |  |
|  |  |  | *Navicula gregaria* Donkin 1861 | NavGre |  |
|  |  |  | *Navicula radiosa* Kützing 1844 | NavRad |  |
|  |  | Neidiaceae |  |  |  |
|  |  |  | *Neidium ampliatum* (Ehrenberg) Krammer 1985 | NeiAmp |  |
|  |  | Pinnulariaceae | |  |  |
|  |  |  | *Pinularia brauniana* (Grunow) Studnicka 1888 | PinBra |  |
|  |  | Sellaporaceae | |  |  |
|  |  |  | *Sellaphora pupula* (Kützing) Mereschkovsky 1902 | SelPup |  |
|  |  |  | *Sellaphora* sp. | SelSp |  |
|  | Cymbellales |  |  |  |  |
|  |  | Cymbellaceae | |  |  |
|  |  |  | *Cymbopleura hustedtii* (Krasske) Novelo, Tavera & Ibarra 2007 | CybHus |  |
|  |  |  | *Cymbella mexicana* (Ehrenberg) Cleve 1894 | CymMex |  |
|  |  | Gomphonemataceae | |  |  |
|  |  |  | *Encyonema* *neogracile* Krammer 1997 | EncNeo |  |
|  |  |  | *Encyonema silesiacum* (Bleisch) Mann 1990 | EncSil |  |
|  |  |  | *Encyonema densistriatum* Novelo, Tavera & Ibarra 2007 | EncDen |  |
|  |  |  | *Encyonema* sp. | EncSp |  |
|  |  |  | *Encyonopsis microcephala* (Grunow) Krammer 1997 | EnyMic |  |
|  |  |  | *Encyonopsis* sp. | EncSp |  |
|  |  |  | *Gomphonema pygmaeum* Kociolek & Stoermer 1991 | GomPyg |  |
|  |  | Rhoicospheniaceae | |  |  |
|  |  |  | *Rhoicosphenia abbreviata* (C.Agardh) Lange-Bertalot 1980 | RhoAbr |  |
|  |  |  |  |  |  |
|  |  |  |  |  |  |
|  |  |  |  |  |  |
|  |  |  |  |  |  |
|  | Mastogloiales | |  |  |  |
|  |  | Mastogloiaceae | |  |  |
|  |  |  | *Mastogloia calcarea* Lee, Gaiser, Van de Vijver, Edlund & Spaulding 2014 | MasCal | ** |
|  |  |  | *Mastogloia elliptica* (Agardh) Cleve 1893 | MasEli | ** |
|  | Bacillariales |  |  |  |  |
|  |  | Bacillariaceae | |  |  |
|  |  |  | *Hantzschia amphioxys* (Ehrenberg) Grunow 1880 | HanAmp |  |
|  |  |  | *Nitzschia acicularis* (Kützing) W.Smith 1853 | NitAsc |  |
|  |  |  | *Nitzschia amphibioides* Hustedt 1942 | NitAmb |  |
|  |  |  | *Nitzschia palea* (Kützing) W.Smith 1856 | NitPal |  |
|  | Rhopalodiales | |  |  |  |
|  |  | Rhopalodiaceae | |  |  |
|  |  |  | *Epithemia reicheltii* Fricke 1904 | EpiRei | ** |
|  | Thalassiophysales |  |  |  |  |
|  |  | Catenulaceae |  |  |  |
|  |  |  | *Amphora copulata* (Kützing) Schoeman & Archibald 1986 | AmpCop |  |
|  |  |  |  |  |  |

** = new record for Mexico according to the Continental Algae Database (bdLACET, Novelo & Tavera, 2021).
