# Supplementary material for: Diatom diversity and distribution in Neotropical karst lakes under anthropogenic stress
Source: PLoS One. 2025 Jul 24;20(7):e0327201. doi: 10.1371/journal.pone.0327201 (PMC12289067; doi:10.1371/journal.pone.0327201)

**S3 Figure. Relative abundances of the diatom taxa present in the studied karstic lakes in southern Mexico.** White bars in *Cyclotella petenensis* represent the percentage of valves showing dissolution. LF = Lacandon region lakes, MBm = Montebello region mountain lakes, MBp = Montebello region plateau lakes. Lakes and species labeled in red correspond to the group of lakes and diatom taxa that had positive CCA1 scores, characteristic of higher salinity, sulphates and trophic conditions.


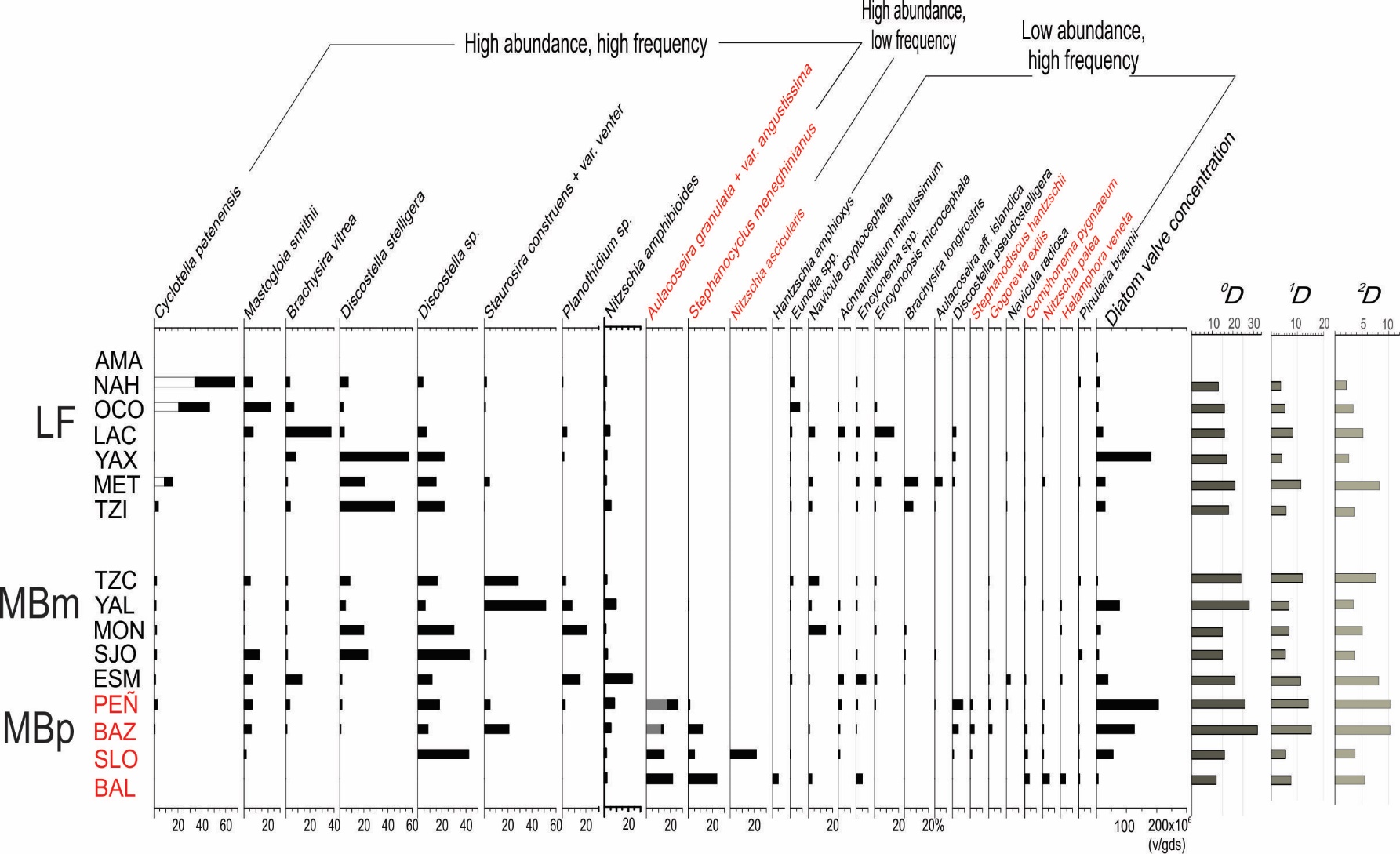

Supplement: S3 Fig — White bars in Cyclotella petenensis represent the percentage of valves showing dissolution. LF = Lacandona region lakes, MBm = Montebello region mountain lakes, MBp = Montebello region plateau lakes. (DOCX) [file pone.0327201.s003.docx]
